# Understanding premenstrual exacerbation: navigating the intersection of the menstrual cycle and psychiatric illnesses

**DOI:** 10.3389/fpsyt.2024.1410813

**Published:** 2024-08-08

**Authors:** Jenny Lin, Christine Nunez, Leah Susser, Liliya Gershengoren

**Affiliations:** ^1^ Department of Psychiatry, New York University, New York, NY, United States; ^2^ Department of Psychiatry, Weill Cornell Medicine, White Plains, NY, United States

**Keywords:** premenstrual exacerbation, premenstrual disorders, women’s mental health, menstrual cycle, mood disorders, psychotic disorders, anxiety disorders, personality disorders

## Abstract

Premenstrual exacerbation of an existing psychiatric disorder refers to the worsening of symptoms inherent to the condition during the premenstrual phase. Research consistently indicates that hormonal fluctuations during the menstrual cycle present a unique period of vulnerability for the onset or exacerbation of psychiatric symptoms, impacting diagnosis, risk assessment, and treatment. This review sought to elucidate the phenomenon of premenstrual exacerbation and its impact across a spectrum of psychiatric illnesses, including mood, anxiety, psychotic, obsessive-compulsive, personality, and trauma-related disorders. Despite the expanded research in recent years on premenstrual dysphoric disorder and premenstrual syndrome, premenstrual exacerbation remains underexplored and poorly defined. This review offers significant contributions to the diagnosis and management of psychiatric conditions, advocating for heightened awareness and novel treatment approaches in the context of premenstrual exacerbation.

## 1 Introduction

There has been a growing recognition of premenstrual symptomatology and premenstrual disorders in recent years, particularly after premenstrual dysphoric disorder (PMDD) was added to the DSM-5 in 2013 (1). Premenstrual symptomatology includes emotional, behavioral, and physical symptoms during the luteal phase that resolve during the onset of menses (2). However, only a subset of these women fulfills the diagnostic criteria for premenstrual disorders, which include PMDD, Premenstrual Syndrome (PMS), and Premenstrual Exacerbation (PME) of underlying psychiatric disorders (1, 3, 4).

While research on PMDD and PMS has expanded, studies focusing on PME remain limited (5). PMDD and PMS are characterized by symptoms that emerge during the late luteal phase and abate following menstruation, with an absence of symptoms during the follicular phase. However, PME is characterized by the worsening of symptoms of another existing disorder during the premenstrual phase, and the symptoms of the existing disorder are still present throughout the entire menstrual cycle (3).

The prevalence of PME in psychiatric disorders remains poorly defined, likely due to the paucity of research and previous studies’ reliance on retrospective assessments, which are prone to inaccuracies. The diagnosis of PME, similar to PMDD and PMS, necessitates prospective symptom tracking across at least two symptomatic menstrual cycles to distinguish it from other conditions and to overcome the limitations of retrospective recall (3).

This review aims to elucidate the relationship between the menstrual cycle and exacerbations of psychiatric illnesses, including mood, anxiety, psychotic, obsessive-compulsive and related disorders, personality disorders, and trauma and stressor-related disorders. Improved understanding of exacerbations of psychiatric illnesses across the menstrual cycle can ultimately improve the diagnosis, risk assessment, and treatment/management in affected individuals.

## 2 Mood disorders

### 2.1 Major depressive disorder

#### 2.1.1 Prevalence

Analyses under the NIMH Sequenced Treatment Alternatives to Relieve Depression (STAR*D) study showed that 64% of premenopausal women with major depressive disorder (MDD) seeking treatment in primary care and psychiatric settings reported premenstrual worsening of their depression (6). This significant figure, however, is derived from retrospective symptom reports, which are susceptible to recall bias, underscoring the necessity for prospective symptom evaluation across at least two symptomatic menstrual cycles for accurate diagnosis of PME. Notably, PME was linked to older age and a higher rate of familial history of depressive disorders and bipolar disorder (7). Aside from specifically PME of MDD, Payne et al. found that more women with MDD are more likely to experience premenstrual or menstrual mood changes compared to those without MDD, highlighting the complex interplay between menstrual cycle phases and mood disorders (8).

#### 2.1.2 Course

Research studies have shown that PME of MDD leads to longer index episodes, more anxiety, and shorter time to relapse after remission (6, 7, 9). The STAR*D trial also showed that women reporting PME were more likely to report physical complaints such as leaden paralysis, gastrointestinal complaints, and psychomotor slowing (6), alongside a greater prevalence of comorbid medical conditions and diminished physical health (7). These women were also less likely to endorse blunted mood reactivity (6).

#### 2.1.3 Treatment

Management of PME in MDD poses unique challenges, as standard antidepressant regimens may not adequately address premenstrual symptom flare-ups. In one small double-blind pilot study, variable dosing of sertraline was found to resolve PME of MDD, with an improvement in difference in scores in depression scales between the luteal and follicular phases when sertraline was increased premenstrually (10). However, the evidence base for this approach remains limited, with few studies supporting the efficacy of premenstrual dosage adjustments in antidepressant therapy (11).

Other studies have also evaluated the efficacy of treatments for PMDD in treating PME of MDD, such as augmentation of antidepressants with combined oral contraceptives or suppression of ovulation with gonadotropin hormone-releasing hormone (GnRH) agonists have had conflicting results (12–14).

### 2.2 Bipolar disorder

#### 2.2.1 Prevalence

In retrospective studies, 64-68% of women reported menstrual cycle-related mood changes, while 44-65% of women in prospective studies reported menstrual cycle-related mood changes (15, 16).

#### 2.2.2 Course

The mood changes associated with the menstrual cycle include depressive symptoms as well as hypomanic or manic symptoms (16). Notably, these fluctuations are not confined to the premenstrual phase but span various stages of the cycle, including ovulation and menstruation (16). Emerging data suggest that premenstrual exacerbation (PME) of bipolar disorder may signal a more challenging disease trajectory, characterized by heightened symptom intensity, reduced intervals to relapse, and increased disruption of daily functioning (16, 17).

There is some evidence to suggest that women are more likely to experience rapid-cycling bipolar disorder, and one hypothesis is that this is due to mood changes secondary to hormonal changes during the menstrual cycle. One study reported that women exhibited large mood fluctuations more frequently than men (15). Rasgon et al. found that a majority of women not taking hormonal contraception reported significant mood changes across the menstrual cycle, though not in any particular direction (17). However, other studies have found no significant change in mood scores across different phases of the menstrual cycle in women with rapid-cycling bipolar disorder (18, 19), or bipolar disorder (20), while others found differences in other phases such as the menstrual phase or follicular phase. These variations make it difficult to understand the pathophysiology behind PME of bipolar disorder.

#### 2.2.3 Treatment

The therapeutic approach to managing PME in bipolar disorder remains underexplored, especially concerning the adjustment of psychotropic medication dosages in response to menstrual cycle phases. However, Robakis et al. found that women taking gamma-aminobutyric acid-A (GABA-A) receptor modulators such as the mood stabilizer lamotrigine had less fluctuation in mood within and across menstrual cycle phases; when combined with hormonal contraception, these medications resulted in improved mood ratings in women with bipolar disorder (21). It is possible that GABA-A receptor modulators could act synergistically with hormonal contraceptives to further target mood symptoms in bipolar disorder (21). There is no empirical data supporting augmentation with an SSRI for treatment of PME in bipolar disorder.

### 2.3 Anxiety disorders

#### 2.3.1 Prevalence

Nillni et al. (22) observed an increase in both the frequency and severity of panic attacks, as well as heightened anxiety and affective symptoms during the premenstrual phase among women with panic disorder (PD), based on retrospective assessments (22). However, when assessed prospectively with daily reports, results were mixed. Some studies reported increased panic attacks and anxiety symptoms during the premenstrual phase, as well as higher Social Avoidance and Distress (SADS) suicidality scores; however, other prospective PD studies found no differences in symptoms across menstrual phases (22–25). Likewise, one study found that women with PD exhibited greater skin conductance levels during anxiety-provoking trials, but no changes in self-reported anxiety when compared to women without PD during the premenstrual phase (26).

Similar mixed patterns have been noted among women with generalized anxiety disorder (GAD) and social anxiety disorder (SAD). Nillni et al. (22) found that approximately 45% of women with GAD retrospectively reported more severe social anxiety and avoidance symptoms during the premenstrual phase, as did women with comorbid GAD and premenstrual syndrome prospectively (24). In contrast, Li et al. (2020) found an increase in repetitive negative thinking (RNT), a key element in the development and maintenance of anxiety, in women with GAD during the mid-luteal phase, but no changes in other GAD symptoms (27). One study found that a subgroup of women with SAD reported more anxiety and avoidance in the premenstrual phase (27, 28). However, findings from prospective studies are more limited, and thus these conclusions are more consistently demonstrated in studies assessing anxiety symptomatology retrospectively (22, 25). Similarly, studies have demonstrated inconsistent findings in cortisol responses following administration of the Trier Social Stress Test (TSST) across menstrual cycle phases (22).

#### 2.3.2 Course

Nillni et al. (22) reports results consistent with prior reviews which theorize that the rapid decline in ovarian hormones could lead to decreased levels of GABAergic neurosteroids, thus altering the anxiolytic function of GABA-A receptors and exacerbating anxiety symptoms premenstrually (22, 25, 27). Another proposed mechanism is that adrenal steroids, such as dehydroepiandrosterone (DHEA), which antagonize GABA-A receptors compete with low levels of allopregnanolone, a progesterone metabolite that normally acts as a positive modulator on the GABA-A receptor, to increase anxiety and post-traumatic stress disorder (PTSD) symptoms (22, 27).

#### 2.3.3 Treatment

Heightened RNT in the luteal phase in women with GAD may correspond with increased susceptibility for the development of or relapse of anxiety disorders. Li et al. (2020) proposed therapy-based interventions to target RNT in women with GAD, such as rumination-focused CBT and traditional CBT. Otherwise, treatment options remain limited for PME of anxiety disorders.

### 2.4 Psychotic disorders

#### 2.4.1 Prevalence

The exacerbation of psychotic disorders with reproductive events such as childbirth has been well-documented, while PME of psychotic disorders has been less well-studied. Gleeson et al. (29) found that 32.4% of women with a schizophrenia-spectrum disorder reported fluctuations of psychotic symptom severity across their cycle (29). Hsiao et al. (24) found that 20% of women fulfilled the definition of PME of schizophrenia (24).

#### 2.4.2 Course

Emerging research suggests a potential role for estrogen in modulating the course of schizophrenia, highlighting gender differences in disease onset, symptomatology, and treatment response. Women tend to exhibit a later disease onset and a higher prevalence of affective symptoms compared to men (29, 30). One hypothesis for these gender differences includes a possible neuroprotective function of estrogen against psychotic symptoms (31), suggesting that low estrogen leads to increased vulnerability to psychosis. Bergemann et al. found that 60% of women with schizophrenia in their study had estradiol serum levels below 30 pg/mL in the follicular phase and below 100 pg/mL in the periovulatory phase (32). Other studies also report that women with schizophrenia have lower estrogen levels compared to normal reference range and also experience menstrual irregularities, though it is difficult to ascertain whether such menstrual irregularities are due to antipsychotic-induced hyperprolactinemia (33, 34).

This effect has been suggested in men as well, with one study demonstrating that estradiol concentrations were inversely correlated with negative symptoms in male patients (35). In addition to this, female schizophrenic patients often have worsening symptoms premenstrually, when estrogen levels are lower (36). Case reports for psychotic symptoms during the premenstrual phase with cessation of symptoms at the onset of menstruation could further support this hypothesis (37). However, this estrogen hypothesis of schizophrenia remains somewhat controversial.

One systematic review and meta-analysis showed that the rate of admissions for women with schizophrenia during the perimenstrual phase was 1.48 times higher than expected (95% CI: 1.31-1.67) (38), suggesting that psychotic symptoms worsen perimenstrually.

#### 2.4.3 Treatment

The treatment of PME in psychotic disorders poses unique challenges. Unlike mood disorders where variable dosing of antidepressants may be beneficial, adjusting doses of antipsychotics, particularly those that raise prolactin levels and subsequently lower estrogen, may not be as straightforward due to the potential exacerbation of symptoms (39). Gattaz et al. (40) found that women who were admitted during phases of the menstrual cycle with low estrogen levels required lower effective doses of antipsychotic medications (40).

Adjunctive estrogen therapy has shown promise in improving outcomes for women with schizophrenia. A randomized, double-blind study comparing women treated with adjunctive transdermal estradiol for 28 days vs. placebo showed that those with adjunctive estradiol had significantly reduced positive and general psychopathological symptoms (41). Similar superiority has been shown with ethinyl estradiol as an adjunct to haloperidol for eight weeks in another double-blind, placebo-controlled trial (42). This effect has also been demonstrated in men, with a randomized placebo-controlled trial in 53 men showing a more rapid reduction in pathology when men with schizophrenia were treated with estradiol as an adjunct to atypical antipsychotic treatment for 14 days, with a reflected increase in serum estrogen levels (43). This effect in men suggests an effect of estrogen on psychopathology rather than an effect related to changes in ovulation. However, other studies have shown variable effects of estrogen supplementation for psychotic symptoms, possibly suggesting an individual variability for response to estrogen supplementation (34, 44, 45).

For women with PME of schizophrenia, adjunctive estradiol therapy premenstrually should be considered and further evaluated. In addition to this, further research should be conducted on the utility of selective estrogen receptor modulators in the treatment of PME of schizophrenia-spectrum disorders.

### 2.5 Personality disorders

#### 2.5.1 Prevalence

Recent research underscores the heightened sensitivity of individuals with borderline personality disorder (BPD) to hormonal fluctuations. Eisenlohr-Moul et al. (46) reported that a significant majority (73%) of unmedicated women with BPD experienced clinically significant PME of emotional symptoms, including but not limited to depression, anxiety, and irritability (46, 47). Notably, the timing of symptom onset varies, with high-arousal symptoms such as irritability and anger intensifying during the luteal phase and peaking premenstrually, whereas low-arousal symptoms like depression emerge closer to menstruation and persist into the follicular phase (46–48). Similarly, a group of undergraduate females with elevated trait BPD features endorsed the most symptomatology during the mid-luteal and perimenstrual phases (46, 47, 49).

#### 2.5.2 Course

Peters et al. (2019) proposes the most likely mechanism for onset of high-arousal symptoms during the luteal phase is an alteration of the GABA-A receptor which reverses the typical positive effects of GABAergic progesterone metabolites such as allopregnanolone, and mimics the onset of PMDD, likely sharing a similar pathophysiology (46, 47). However, low-arousal symptoms of BPD demonstrated PME with later onset in the menstrual cycle (MC) and more prolonged elevation, which is unlike the rapid resolution of PMDD symptoms in conjunction with the follicular phase, likely suggesting a different mechanism of action (46, 47). Another proposed theory for PME of low-arousal symptoms is that women with BPD may have greater serotonergic sensitivity to fluctuations in ovarian steroid hormones (47) In addition, studies have observed a greater proportion of and more lethal suicide attempts occurring in the early follicular phase, when ovarian steroids are at their lowest (47).

#### 2.5.3 Treatment

To date, no pharmacological interventions have been specifically designed to target PME in BPD. However, the use of SSRIs during the luteal phase has demonstrated reduction of some PMDD symptoms, suggesting potential benefits for managing high-arousal symptoms in BPD (47, 48). Although studies have shown oral contraceptives to be beneficial for PMDD, they have been found to worsen symptoms in women with BPD and warrant further research (47, 48). Other studies have preliminarily demonstrated that stabilization of ovarian steroid hormones in a sample of women with suicidal ideation reduced PME of low-arousal symptoms (47, 48). Finally, non-pharmacological interventions can also be implemented such as cycle-tracking to increase awareness and develop appropriate coping strategies, ideally being combined with therapy modalities such as dialectical behavior therapy (DBT) (46–48).

### 2.6 Obsessive-compulsive disorder and related disorders

#### 2.6.1 Prevalence

PME of obsessive-compulsive disorder (OCD) has been retrospectively reported in 20-42% of women (47, 50). One prospective study found that there was an increase in symptoms during the premenstrual phase compared to mid-cycle (50).

#### 2.6.2 Course

The severity of OCD symptoms has been linked to reproductive cycle events characterized by low estrogen levels, such as the postpartum period and menarche (50). Forray et al. found that women who experienced OCD onset in the perinatal period and perinatal worsening of OCD were more likely to have PME of OCD symptoms compared to those who denied OCD onset related to pregnancy (65% vs. 39.3%, p = 0.047) (51). This phenomenon may be underpinned by serotonergic dysregulation, exacerbated by the hormonal shifts of the menstrual cycle (52, 53).

There is also limited evidence to suggest that OCD symptoms fluctuate throughout the menstrual cycle (54). Women with PME of obsessive-compulsive symptoms had a statistically higher frequency of suicidal ideation, suicide attempts, and higher scores on the Beck Depression Inventory (55). These women were also more likely to have current use of selective serotonin reuptake inhibitors (SSRI), lifetime use of mood stabilizers, and sexual/religious obsessions (55).

#### 2.6.3 Treatment

The limited evidence available underscores a significant gap in our understanding and management of menstrual cycle-related symptom fluctuations in OCD.

### 2.7 Trauma and stressor-related disorders

#### 2.7.1 Prevalence

Research on PME of trauma and stressor-related disorders is limited. Preliminary reports suggest an increase in PTSD symptoms during the premenstrual and menstrual phases (56).

#### 2.7.2 Course

Emerging research indicates that women with PTSD may experience heightened fear-related and avoidance symptoms premenstrually and during menstruation (56). Studies have also shown that intrusive flashbacks could be exacerbated during different phases of the menstrual cycle, though studies show differing phases where flashbacks were worse (56–58).

#### 2.7.3 Treatment

Studies evaluating the treatment of possible PME of trauma-related disorders symptoms remain limited.

## 3 Discussion

This review has sought to elucidate the phenomenon of PME and its impact across a spectrum of psychiatric illnesses, including mood, anxiety, psychotic, obsessive-compulsive disorders, personality disorders, and trauma-related disorders. It emerges that a subset of women exhibits a heightened sensitivity to the normal fluctuations of ovarian steroid hormones, leading to PME of symptoms inherent to their psychiatric condition. Research consistently indicates that hormonal fluctuations during the MC present a unique period of vulnerability for the onset or exacerbation of psychiatric symptoms. Notably, PME appears to affect certain disorders more profoundly, suggesting the involvement of distinct neurological and psychological mechanisms (25, 46). Despite these findings, the impact of menstrual cycle-induced hormonal changes on the diagnosis and management of psychiatric disorders remains largely underappreciated (25).

### 3.1 Limitations

There are numerous limitations that hinder the ability to conduct robust research on the prevalence of PME of pre-existing psychiatric disorders (25). Many current studies rely on the use of retrospective assessment of PME which is limited by participants’ memory and potential biased recall. The prospective assessment of symptoms is considered the gold standard, via data collection throughout the entirety of the MC (25, 27). In addition, there are potential effect modifiers that may be difficult to completely eliminate: the effect of psychotropic medication on hormonal fluctuations as well as the role of the MC on drug absorption and metabolization, usage of hormonal contraception, and inpatient treatment settings which may bias towards a treatment effect (25). Therefore, study results should be interpreted in light of these modifiers. Future studies should aim to implement standardized menstrual phase definitions, hormonal assays to verify cycle phase, a minimum observation period of three menstrual cycles, prospective symptom measurement, characterization of participants’ reproductive status (naturally cycling, pregnant, menopausal), and larger sample sizes (25, 27). Future areas of study include the role of testosterone in premenstrual exacerbation of psychiatric disorders as well as randomized trials clarifying the efficacy of hormonal treatments and contraceptives for PME of psychiatric disorders.

### 3.2 Implications

Overall, there remains a lack of robust evidence into PME of known psychiatric disorders. Recognizing PME is crucial, as fluctuations in symptom severity related to the MC can significantly affect diagnosis, risk assessment, and treatment planning (25). The persistence of symptom exacerbation throughout the MC, despite pharmacological intervention, calls for a reevaluation of existing treatment paradigms (25). Preliminary findings suggest that certain disorders, particularly mood disorders and borderline personality disorder, exhibit a higher sensitivity to PME, warranting more frequent screening and tailored management strategies (25). Finally, psychopharmacologic and psychosocial treatment modalities to address PME are vastly understudied, but many existing interventions such as CBT and DBT may be used to target certain symptoms (47).

## Author contributions

JL: Conceptualization, Writing – original draft, Writing – review & editing. CN: Conceptualization, Writing – original draft, Writing – review & editing. LS: Writing – review & editing. LG: Writing – original draft, Writing – review & editing.

## References

[1] American Psychiatric Association. Diagnostic and statistical manual of mental disorders, 5th ed. Washington DC: American Psychiatric Association. (2013). doi: 10.1176/appi.books.9780890425596.

[2] Yonkers KA O'Brien PM Eriksson E. Premenstrual syndrome. Lancet. (2008) 371:1200–10. doi: 10.1016/S0140-6736(08)60527-9 PMC3118460 18395582

[3] O'Brien PM Bäckström T Brown C Dennerstein L Endicott J Epperson CN. Towards a consensus on diagnostic criteria, measurement and trial design of the premenstrual disorders: the ISPMD Montreal consensus. Arch Womens Ment Health. (2011) 14:13–21. doi: 10.1007/s00737-010-0201-3 21225438 PMC4134928

[4] Hofmeister S Bodden S. Premenstrual syndrome and premenstrual dysphoric disorder. Am Fam Physician. (2016) 94:236–40. 27479626

[5] Tiranini L Nappi RE. Recent advances in understanding/management of premenstrual dysphoric disorder/premenstrual syndrome. Fac Rev. (2022) 11:11. doi: 10.12703/r 35574174 PMC9066446

[6] Kornstein SG Harvey AT Rush AJ Wisniewski SR Trivedi MH Svikis DS. Self-reported premenstrual exacerbation of depressive symptoms in patients seeking treatment for major depression. Psychol Med. (2005) 35:683–92. doi: 10.1017/S0033291704004106 15918345

[7] Haley CL Sung SC Rush AJ Trivedi MH Wisniewski SR Luther JF. The clinical relevance of self-reported premenstrual worsening of depressive symptoms in the management of depressed outpatients: a STAR*D report. J Womens Health (Larchmt). (2013) 22:219–29. doi: 10.1089/jwh.2011.3186 PMC3634137 23480315

[8] Payne JL Roy PS Murphy-Eberenz K Weismann MM Swartz KL McInnis MG. Reproductive cycle-associated mood symptoms in women with major depression and bipolar disorder. J Affect Disord. (2007) 99:221–9. doi: 10.1016/j.jad.2006.08.013 17011632

[9] Knezević G Opacić G Savić D Priebe S. Do personality traits predict post-traumatic stress?: a prospective study in civilians experiencing air attacks. Psychol Med. (2005) 35:659–63. doi: 10.1017/s0033291704004131 15918342

[10] Miller MN Newell CL Miller BE Frizzell PG Kayser RA Ferslew KE. Variable dosing of sertraline for premenstrual exacerbation of depression: a pilot study. J Womens Health (Larchmt). (2008) 17:993–7. doi: 10.1089/jwh.2007.0491 18681820

[11] Miller MN Miller BE Chinouth R Coyle BR Brown GR. Increased premenstrual dosing of nefazodone relieves premenstrual magnification of depression. Depress Anxiety. (2002) 15:48–51. doi: 10.1002/(ISSN)1520-6394 11816054

[12] Freeman EW Sondheimer SJ Rickels K. Gonadotropin-releasing hormone agonist in the treatment of premenstrual symptoms with and without ongoing dysphoria: a controlled study. Psychopharmacol Bull. (1997) 33:303–9. 9230648

[13] Joffe H Petrillo LF Viguera AC Gottshcall H Soares CN Hall JE. Treatment of premenstrual worsening of depression with adjunctive oral contraceptive pills: a preliminary report. J Clin Psychiatry. (2007) 68:1954–62. doi: 10.4088/JCP.v68n1218 18162029

[14] Peters W Freeman MP Kim S Cohen LS Joffe H. Treatment of premenstrual breakthrough of depression with adjunctive oral contraceptive pills compared with placebo. J Clin Psychopharmacol. (2017) 37:609–14. doi: 10.1097/JCP.0000000000000761 28816924

[15] Rasgon N Bauer M Grof P Gyulai L Elman S Glenn T. Sex-specific self-reported mood changes by patients with bipolar disorder. J Psychiatr Res. (2005) 39:77–83. doi: 10.1016/j.jpsychires.2004.05.006 15504425

[16] Teatero ML Mazmanian D Sharma V. Effects of the menstrual cycle on bipolar disorder. Bipolar Disord. (2014) 16:22–36. doi: 10.1111/bdi.12138 24467469

[17] Dias RS Lafer B Russo C Del Debbio A Nierenberg AA Sachs GS. Longitudinal follow-up of bipolar disorder in women with premenstrual exacerbation: findings from STEP-BD. Am J Psychiatry. (2011) 168:386–94. doi: 10.1176/appi.ajp.2010.09121816 21324951

[18] Leibenluft E Ashman SB Feldman-Naim S Yonkers KA. Lack of relationship between menstrual cycle phase and mood in a sample of women with rapid cycling bipolar disorder. Biol Psychiatry. (1999) 46:577–80. doi: 10.1016/S0006-3223(99)00023-2 10459410

[19] Shivakumar G Bernstein IH Suppes T Keck PE McElroy SL Altshuler LL. Are bipolar mood symptoms affected by the phase of the menstrual cycle? J Womens Health (Larchmt). (2008) 17:473–8. doi: 10.1089/jwh.2007.0466 18328012

[20] Sit D Seltman H Wisner KL. Menstrual effects on mood symptoms in treated women with bipolar disorder. Bipolar Disord. (2011) 13:310–7. doi: 10.1111/bdi.2011.13.issue-3 PMC3117221 21676134

[21] Robakis TK Holtzman J Stemmle PG Reynolds-May MF Kenna HA Rasgon NL. Lamotrigine and GABAA receptor modulators interact with menstrual cycle phase and oral contraceptives to regulate mood in women with bipolar disorder. J Affect Disord. (2015) 175:108–15. doi: 10.1016/j.jad.2014.12.040 PMC4352404 25601310

[22] Nillni YI Rasmusson AM Paul EL Pineles SL. The impact of the menstrual cycle and underlying hormones in anxiety and PTSD: what do we know and where do we go from here? Curr Psychiatry Rep. (2021) 23:8. doi: 10.1007/s11920-020-01221-9 33404887 PMC8819663

[23] Başoğlu C Cetin M Semiz UB Ağargün MY Ebrinç S. Premenstrual exacerbation and suicidal behavior in patients with panic disorder. Compr Psychiatry. (2000) 41:103–5. doi: 10.1016/S0010-440X(00)90141-X 10741887

[24] Hsiao MC Hsiao CC Liu CY. Premenstrual symptoms and premenstrual exacerbation in patients with psychiatric disorders. Psychiatry Clin Neurosci. (2004) 58:186–90. doi: 10.1111/j.1440-1819.2003.01215.x 15009825

[25] Nolan LN Hughes L. Premenstrual exacerbation of mental health disorders: a systematic review of prospective studies. Arch Womens Ment Health. (2022) 25:831–52. doi: 10.1007/s00737-022-01246-4 35867164

[26] Sigmon ST Dorhofer DM Rohan KJ Hotovy LA Boulard NE Fink CM. Psychophysiological, somatic, and affective changes across the menstrual cycle in women with panic disorder. J Consult Clin Psychol. (2000) 68:425–31. doi: 10.1037/0022-006X.68.3.425 10883559

[27] Li S. H. Denson T. F. Graham B. M. Women with generalized anxiety disorder show increased repetitive negative thinking during the luteal phase of the menstrual cycle. Clin Psychological Science. (2020) 8 (6):1037–45. doi: 10.1177/2167702620929635

[28] van Veen JF Jonker BW van Vliet IM Zitman FG. The effects of female reproductive hormones in generalized social anxiety disorder. Int J Psychiatry Med. (2009) 39:283–95. doi: 10.2190/PM.39.3.e 19967900

[29] Gleeson PC Worsley R Gavrilidis E Nathoo S Ng E Lee S. Menstrual cycle characteristics in women with persistent schizophrenia. Aust N Z J Psychiatry. (2016) 50:481–7. doi: 10.1177/0004867415590459 26070315

[30] Li X Zhou W Yi Z. A glimpse of gender differences in schizophrenia. Gen Psychiatr. (2022) 35:e100823. doi: 10.1136/gpsych-2022-100823 36118418 PMC9438004

[31] Gogos A Sbisa AM Sun J Gibbons A Udawela M Dean B. A role for estrogen in schizophrenia: clinical and preclinical findings. Int J Endocrinol. (2015) 2015:615356. doi: 10.1155/2015/615356 26491441 PMC4600562

[32] Bergemann N Mundt C Parzer P Jannakos I Nagl I Salbach B. Plasma concentrations of estradiol in women suffering from schizophrenia treated with conventional versus atypical antipsychotics. Schizophr Res. (2005) 73:357–66. doi: 10.1016/j.schres.2004.06.013 15653282

[33] Huber TJ Rollnik J Wilhelms J von zur Mühlen A Emrich HM Schneider U. Estradiol levels in psychotic disorders. Psychoneuroendocrinology. (2001) 26:27–35. doi: 10.1016/S0306-4530(00)00034-2 11070332

[34] Bergemann N Mundt C Parzer P Pakrasi M Eckstein-Mannsperger U Haisch S. Estrogen as an adjuvant therapy to antipsychotics does not prevent relapse in women suffering from schizophrenia: results of a placebo-controlled double-blind study. Schizophr Res. (2005) 74:125–34. doi: 10.1016/j.schres.2004.12.009 15721993

[35] Kaneda Y Ohmori T. Relation between estradiol and negative symptoms in men with schizophrenia. J Neuropsychiatry Clin Neurosci. (2005) 17:239–42. doi: 10.1176/jnp.17.2.239 15939980

[36] Grigoriadis S Seeman MV. The role of estrogen in schizophrenia: implications for schizophrenia practice guidelines for women. Can J Psychiatry. (2002) 47:437–42. doi: 10.1177/070674370204700504 12085678

[37] Ahern E Cohen D Prior C Raji E. Menstrual psychosis. Ir J Psychol Med. (2022) 39:103–5. doi: 10.1017/ipm.2019.36 31500681

[38] Reilly TJ Sagnay de la Bastida VC Joyce DW Cullen AE McGuire P. Exacerbation of psychosis during the perimenstrual phase of the menstrual cycle: systematic review and meta-analysis. Schizophr Bull. (2020) 46:78–90. doi: 10.1093/schbul/sbz030 31071226 PMC6942155

[39] Seeman MV. Menstrual exacerbation of schizophrenia symptoms. Acta Psychiatr Scand. (2012) 125:363–71. doi: 10.1111/j.1600-0447.2011.01822.x 22235755

[40] Gattaz WF Vogel P Riecher-Rössler A Soddu G. Influence of the menstrual cycle phase on the therapeutic response in schizophrenia. Biol Psychiatry. (1994) 36:137–9. doi: 10.1016/0006-3223(94)91195-9 7948447

[41] Kulkarni J de Castella A Fitzgerald PB Gurvich CT Bailey M Bartholomeusz C. Estrogen in severe mental illness: a potential new treatment approach. Arch Gen Psychiatry. (2008) 65:955–60. doi: 10.1001/archpsyc.65.8.955 18678800

[42] Akhondzadeh S Nejatisafa AA Amini H Mohammadi MR Larijani B Kashani L. Adjunctive estrogen treatment in women with chronic schizophrenia: a double-blind, randomized, and placebo-controlled trial. Prog Neuropsychopharmacol Biol Psychiatry. (2003) 27:1007–12. doi: 10.1016/S0278-5846(03)00161-1 14499318

[43] Kulkarni J de Castella A Headey B Marston N Sinclair K Lee S. Estrogens and men with schizophrenia: is there a case for adjunctive therapy? Schizophr Res. (2011) 125:278–83. doi: 10.1016/j.schres.2010.10.009 21062669

[44] Liao DL Chen H Lee SM Tsai SJ. Estrogen supplementation for female schizophrenics treated with atypical antipsychotics. Gen Hosp Psychiatry. (2002) 24:357–9. doi: 10.1016/S0163-8343(02)00193-7 12220803

[45] Louzã MR Marques AP Elkis H Bassitt D Diegoli M Gattaz WF. Conjugated estrogens as adjuvant therapy in the treatment of acute schizophrenia: a double-blind study. Schizophr Res. (2004) 66:97–100. doi: 10.1016/S0920-9964(03)00082-3 15061241

[46] Eisenlohr-Moul TA Schmalenberger KM Owens SA Peters JR Dawson DN Girdler SS. Perimenstrual exacerbation of symptoms in borderline personality disorder: evidence from multilevel models and the Carolina Premenstrual Assessment Scoring System. Psychol Med. (2018) 48:2085–95. doi: 10.1017/S0033291718001253 PMC6436806 29804553

[47] Peters JR Eisenlohr-Moul TA. Ovarian hormones as a source of fluctuating biological vulnerability in borderline personality disorder. Curr Psychiatry Rep. (2019) 21:109. doi: 10.1007/s11920-019-1096-y 31624929 PMC7047501

[48] Peters JR Owens SA Schmalenberger KM Eisenlohr-Moul TA. Differential effects of the menstrual cycle on reactive and proactive aggression in borderline personality disorder. Aggress Behav. (2020) 46:151–61. doi: 10.1002/ab.21877 PMC7458153 31957896

[49] Eisenlohr-Moul TA DeWall CN Girdler SS Segerstrom SC. Ovarian hormones and borderline personality disorder features: Preliminary evidence for interactive effects of estradiol and progesterone. Biol Psychol. (2015) 109:37–52. doi: 10.1016/j.biopsycho.2015.03.016 25837710 PMC4516641

[50] Labad J Menchón JM Alonso P Segalàs C Jiménez S Vallejo J. Female reproductive cycle and obsessive-compulsive disorder. J Clin Psychiatry. (2005) 66:428–35. doi: 10.4088/JCP.v66n0404 15816784

[51] Forray A Focseneanu M Pittman B McDougle CJ Epperson CN. Onset and exacerbation of obsessive-compulsive disorder in pregnancy and the postpartum period. J Clin Psychiatry. (2010) 71:1061–8. doi: 10.4088/JCP.09m05381blu PMC4204467 20492843

[52] Jenike MA Rauch SL Cummings JL Savage CR Goodman WK. Recent developments in neurobiology of obsessive-compulsive disorder. J Clin Psychiatry. (1996) 57:492–503. doi: 10.4088/JCP.v57n1009 8909341

[53] Steiner M Dunn E Born L. Hormones and mood: from menarche to menopause and beyond. J Affect Disord. (2003) 74:67–83. doi: 10.1016/S0165-0327(02)00432-9 12646300

[54] Vulink NC Denys D Bus L Westenberg HG. Female hormones affect symptom severity in obsessive-compulsive disorder. Int Clin Psychopharmacol. (2006) 21:171–5. doi: 10.1097/01.yic.0000199454.62423.99 16528139

[55] Moreira L Bins H Toressan R Ferro C Harttmann T Petribú K. An exploratory dimensional approach to premenstrual manifestation of obsessive-compulsive disorder symptoms: a multicentre study. J Psychosom Res. (2013) 74:313–9. doi: 10.1016/j.jpsychores.2012.12.004 23497833

[56] Nillni YI Pineles SL Patton SC Rouse MH Sawyer AT Rasmusson AM. Menstrual cycle effects on psychological symptoms in women with PTSD. J Trauma Stress. (2015) 28:1–7. doi: 10.1002/jts.21984 25613589

[57] Bryant RA Felmingham KL Silove D Creamer M O'Donnell M McFarlane AC. The association between menstrual cycle and traumatic memories. J Affect Disord. (2011) 131:398–401. doi: 10.1016/j.jad.2010.10.049 21093927

[58] Ferree NK Kamat R Cahill L. Influences of menstrual cycle position and sex hormone levels on spontaneous intrusive recollections following emotional stimuli. Conscious Cognit. (2011) 20:1154–62. doi: 10.1016/j.concog.2011.02.003 PMC3126908 21353599

